# Palliative care of proximal femur metastatic disease and osteolytic lesions: results following surgical and radiation treatment

**DOI:** 10.1186/s12885-024-13170-0

**Published:** 2024-11-21

**Authors:** Elisabeth Mehnert, Fränze Sophie Möller, Christine Hofbauer, Anne Weidlich, Doreen Winkler, Esther G. C. Troost, Christina Jentsch, Konrad Kamin, Marcel Mäder, Klaus-Dieter Schaser, Hagen Fritzsche

**Affiliations:** 1 University Center of Orthopedic, Trauma and Plastic Surgery, Faculty of Medicine, University Hospital Carl Gustav Carus, Technische Universität Dresden, Dresden, Germany; 2 National Center for Tumor Diseases (NCT/UCC), Partner Site Dresden, Germany: German Cancer Research Center (DKFZ), Heidelberg, Germany; 3 Faculty of Medecine and University Hospital Carl Gustav Carus, Technische Universität Dresden, Helmholtz-Zentrum Dresden-Rossendorf, Dresden, Germany; 4 German Cancer Consortium (DKTK), Partner Site Dresden, and German Cancer Research Center (DKFZ), Heidelberg, Germany; 5 Department of Radiotherapy and Radiation Oncology, Faculty of Medicine and University Hospital Carl Gustav Carus, Technische Universität Dresden, Dresden, Germany; 6 OncoRay – National Center for Radiation Research in Oncology, Faculty of Medicine and University Hospital Carl Gustav Carus, Technische Universität Dresden, Helmholtz-Zentrum Dresden-Rossendorf, Dresden, Germany; 7 Helmholtz-Zentrum Dresden-Rossendorf, Institute of Radiooncology – OncoRay, Dresden, Germany

**Keywords:** Proximal femoral metastasis, Osteolytic bone lesions, Pathological fracture, Impending fracture, Intramedullary nailing, Multimodal cancer therapies, Skeletal-related events

## Abstract

**Background:**

Femoral bone metastases (FBM) or lesions (FBL) can lead to loss of mobility and independence due to skeletal-related events (SRE), e.g. pain, deformity and pathological fractures. Aim of this study was to analyze effects of radiotherapy and surgery, different surgical techniques and complications on disease-specific survival (DSS).

**Methods:**

Patients who underwent palliative therapy for FBM or FBL between 2014 and 2020 were retrospectively analyzed. Chi-square test was used to detect intergroup differences. Survival was calculated using Kaplan-Meier method, Cox regression and compared using log-rank test. Complications were evaluated using Chi-Square test.

**Results:**

145 patients were treated for proximal femoral BM/BL or pathologic fractures (10 bilaterally). Three groups were classified: surgery only (S, *n* = 53), surgery with adjuvant radiation (S + RT, *n* = 58), and primary radiation only (RT, *n* = 44). Most common primary tumors were breast (*n* = 31), prostate (*n* = 27), and non-small cell lung cancer (*n* = 27). 47 patients underwent surgery for an impending, 61 for a manifest pathological fracture. There were no significant differences in DSS between the 3 groups (S = 29.8, S + RT = 32.2, RT = 27.1 months), with the S + RT group having the longest one-year survival. Local complications occurred in 25 of 145 patients after a mean interval of 9.9 months.

**Conclusion:**

Due to the steadily increasing incidence and survival of patients with FBM/FBL, indication for prevention and treatment of painful and immobilizing SREs should be critically assessed. Surgical treatment should always be performed with maximum stability and, whenever possible, adjuvant RT.

## Introduction

The number of patients suffering from tumor diseases is steadily increasing [1]. Due to constantly improving therapies, more and more patients are showing longer disease-specific survival (DSS) despite advanced metastatic tumor stages. Statistically, most skeletal malignancies in adults over 40 years of age do not originate from primary bone tumors, but are metastases or bone lesions secondary to carcinomas, multiple myelomas or lymphomas [2, 3].

Underlying malignancies with osteotropic pattern of skeletal metastasis or manifestation typically include breast, prostate, renal, lung and thyroid cancers as well as multiple myeloma, respectively [2, 4, 5]. Many patients develop complications from advanced skeletal metastasis, which are referred to as skeletal-related events (SREs). These include pain, pathological fractures, deformities, neurovascular impairment (e.g. spinal cord compression) and usually require radiotherapy and / or surgery [6]. The loss of mobility and the associated limited participation in social life not only lead to a significant reduction in quality of life [6, 7]. The treatment of skeletal metastases depends on patient-specific (age and general health as well as stage/extension/localization of the disease and tumor-specific (tumor entity, symptoms and response to radio-/ polychemotherapies) factors [2, 8]. The proximal femur is the most frequently affected metastatic site of the appendicular skeleton [4, 9]. Prophylactic stabilization in palliative treatment can be performed to prevent metastatic pathological fractures, which are known to not only compromise stability but also -when operated- are associated with increased blood loss, poor healing and outcome. By avoiding proximal femoral fractures, patients mortality can be reduced by up to 25% [3, 10]. There are scoring systems to assist decision making if prophylactic fixation is necessary, among which the Mirels Score [11, 12] is most often used, considering localisation, local extent and type (osteolytic vs. osteoblastic) as well as intensity of the associated pain. With a score of 7–9, prophylactic stabilization is relatively recommended, > 9 absolutely indicated [9]. Depending on the location at the proximal femur, metastases and pathological fractures are mostly fixed by cephalomedullary interlocking nails, hemi- or total endoprosthetic replacement [3, 13]. Pain reduction and preservation or restoration of function are the most important goals of surgical therapy [14]. Metastatic bone pain can also be reliably relieved by radiation therapy alone, but the onset of effect may take 4 to 12 weeks [15]. Due to the slow or non-healing nature of pathological fractures and the limited individual life expectancy, sufficient, ultimate and safe weight-bearing reconstructions with a very low complication profile are essential for these patients. In addition, adjuvant local radiotherapy can reduce pain and possibly lower the risk of local tumor progression [3, 16, 17]. Currently there is no consensus for the treatment of patients with unstable proximal femoral metastases or pathological femoral fractures.

The aim of this study was to retrospectively analyse the individual and combined influence of local surgical and radiation treatment on DSS and the occurrence of postoperative/interventional complications in patients with stability-threatening proximal femoral metastases/lesions and pathological proximal femoral fractures. In addition, the influence of the underlying tumor entities as well as the presence of visceral, pulmonary, lymphogenous and cerebral metastasis, especially in comparison to osseous metastasis alone, should be investigated with regard to DSS.

## Materials and methods

This retrospective study included patients who received local therapy for metastases, osseous lesions of hematopoietic system diseases or metastatic-induced pathological fractures of the proximal femur between 2014 and 2020 in palliative intent. This retrospective analysis was approved by the Ethics Committee of the Technische Universität Dresden, Germany (BO-EK-73022021). Data were obtained from the electronic patient file ORBIS (Dedalus HealthCare, Bonn, Germany) and the tumor documentation system of the NCT/UCC Dresden. Identification was based on ICD-10 coding. The ICD-10 codes considered were C79.5, M90.75 and M89.55. The resulting patient data were then used to identify patients who had been treated for a metastasis of the proximal femur. Bone metastasis was confirmed using X-ray, CT, MRI, PET-CT or -MRI. Patients with a solitary primary bone tumor or a solitary metastasis of carcinoma were excluded. Patients with palliative treatment due to metastatic carcinoma, bone and soft tissue sarcoma, as well as osseous lesions of haematological neoplasia were included in the study. The decision on the indication for local therapy (S, S + RT or RT) was usually made in the interdisciplinary tumor board, in which a surgeon and radiotherapist participated. However, the study also included patients who had been referred by external hospitals or outpatient providers either exclusively for local therapy or did not undergo postoperative radiation despite it was clearly recommended. Very few patients were not always consistently presented at our interdisciplinary tumor board (2–3 times held per week) due to extraordinary pain syndrome and emergency indication for surgical fixation. All these factors clearly limit the quality and representativity of our findings, however, on occasion are difficult to manage and not always avoidable. The indications for total treatment, surgery and radiation were divided into 7 subgroups according to the localization of metastasis within the proximal femur region, i.e. epiphysis, cervical neck or sub-/petrochanteric (Table 1). The patients’ performance status was evaluated using the ECOG (Eastern Cooperative Oncology Group; Assessment of the health condition of patients) status.

**Table 1:** Patient and tumor characteristics (*significant in the 95% confidence interval)

		All *n* = 155 (%)	S *n* = 53 (%)	S + RT *n* = 58 (%)	RT *n* = 44 (%)	Chi-square test (*p* =)
Sex	Female	74 (47.7)	31(58.5)	27 (46.6)	16 (36.4)	0.92
	Male	81 (52.3)	22 (41.5)	31 (53.4)	28 (63.6)	
Age			71 (20–99)	69.2 (28–90)	71.2 (49–94)	0.397
ECOG	0	15 (9.7)	4 (7.5)	7 (12.1)	4 (9.1)	< 0.001*
	1	63 (40.6)	23 (43.4)	29 (50.0)	11 (25.0)	
	2	43 (27.7)	21 (39.6)	14 (24.1)	8 (18.2)	
	3	16 (10.3)	4 (7.5)	8 (13.8)	4 (9.1)	
	4	1 (0.6)	1 (1.9)			
	Not specified	17 (11.0)			17 (38.6)	
Histology	Breast carcinoma	34 (21.9)	12 (22.6)	16 (27.6)	6 (13.6)	0.071
	Prostate carcinoma	28 (18.1)	6 (11.3)	9 (15.5)	13 (29.5)	
	NSCLC	27 (17.4)	9 (17.0)	7 (12.1)	11 (25.0)	
	Renal cell carcinoma	15 (9.7)	5 (9.4)	7 (12.1)	3 (6.8)	
	Multiple myeloma	17 (11)	8 (15.1)	8 (13.8)	1 (2.3)	
	CUP^1^ adenocarcinoma	6 (3.9)	1 (1.9)	4 (6.9)	1 (2.3)	
	Urothelial carcinoma	3 (1.9)		2 (3.4)	1 (2.3)	
	Esophageal carcinoma	3 (1.9)	1 (1.9)	1 (1.7)	1 (2.3)	
	Rectal adenocarcinoma	3 (1.9)	2 (3.8)	1 (1.7)		
	Rhabdomyosarcoma	2 (1.3)	2 (3.8)			
	SCLC	2 (1.3)			2 (4.5)	
	Angiosarcoma	2 (1.3)		1 (1.7)	1 (2.3)	
	B-cell lymphoma	2 (1.3)	2 (3.8)			
	Colon adenocarcinoma	2 (1.3)	2 (3.8)			
	Hepatocellular carcinoma	2 (1.3)			2 (4.5)	
	GIST^2^	1 (0.6)			1 (2.3)	
	Thyroid carcinoma	1 (0.6)			1 (2.3)	
	Laryngeal squamous cell carcinoma	1 (0.6)	1 (1.9)			
	Leiomyosarcoma	1 (0.6)		1 (1.7)		
	CLL^3^	1 (0.6)	1 (1.9)			
	Malignant melanoma	1 (0.6)		1 (1.7)		
	Penile squamous cell carcinoma	1 (0.6)	1 (1.9)			
Multimodal therapy	Yes	100 (64.5)	26 (49.1)	44 (75.9)	30 (68.2)	0.117
	No	49 (31.6)	23 (43.4)	14 (24.1)	12 (27.3)	
	Not specified	6 (3.9)	4 (7.5)		2 (4.5)	
Treatment indication	Medial femoral neck fracture	37 (23.9)	26 (49.1)	8 (13.8)	3 (6.8)	< 0.001*
	Subtrochanteric femoral fracture	17 (11.0)	10 (18.9)	7 (12.1)		
	Intertrochanteric femoral fracture	11 (7.1)	5 (9.4)	6 (10.3)		
	Intertrochanteric metastasis/lesions	43 (27.7)	9 (17.0)	20 (34.5)	14 (31.8)	
	Femoral neck metastasis/lesions	28 (18.1)	3 (5.7)	11 (19.0)	14 (31.8)	
	Subtrochanteric metastasis/lesions	12 (7.7)		6 (10.3)	6 (13.6)	
	Femoral head metastasis/lesions	7 (4.5)			7 (15.9)	
Implant	Proximal femoral nail PFNA Gamma nail Targon® nail	68 (43.9) 60 (38.7) 4 (2.6) 4 (2.6)	23 (43.4) 19 (35.8) 2 (3.8) 2 (3.8)	45 (77.6) 41 (70.7) 2 (3.4) 2 (3.4)		0.053
	Dynamic hip screw	3 (1.9)	1 (1.9)	2 (3.4)		
	Total hip arthroplasty	19 (34.5)	13 (24.5)	6 (10.3)		
	Hemiarthroplasty	17 (11.0)	12 (22.6)	5 (8.6)		
	Proximal femoral replacement	2 (1.3)	2 (3.8)			
Mirels Score		9,7 ± 1,1	9,9 ± 1,1	9,6 ± 1,1	8,1 ± 1,3	< 0.001*

*Abbreviations *^1^Cancer of unknown primary, ^2^ Gastrointestinal stromal tumor, ^3^ Chronic lymphocytic leukemia, AWD = alive with disease, DOD = died of disease, DOC = died of complications, DOO = died of other circumstances

Statistical analysis was performed using SPSS software version 28.0.0.0 (SPSS. Inc., Chicago, IL). DSS was defined as time between initial diagnosis of malignancy and death due to the cancer disease. DSS was calculated using Kaplan-Meier methodology and compared by log-rank test and Cox regression analysis. Values are presented as mean, minimum/maximum or 95% confidence interval (mean, minimum – maximum, 95% CI). For detection of differences between two or more groups, chi-square test was used. To analyse inter-group differences in independent variables, we conducted a Mann-Whitney-U-Test. Prior to this, the data was tested for normal distribution using the Shapiro-Wilk-Test. The level of significance was *p* < 0.05 regarding all statistical tests.

## Results

A total of 145 patients were identified, of whom 10 patients were treated bilaterally (eight operated, two only locally irradiated). A total of 76 male (81 cases) and 69 female patients (74 cases) underwent local treatment. The left and right sides were affected in 77 and 78 cases, respectively.

A total of 22 different underlying primary tumor entities (14 carcinoma / 3 sarcoma / 3 hematopoietic neoplasms / 1 x melanoma) were identified. An overview of clinical characteristics is shown in Table 1.

**Fig. 1:**
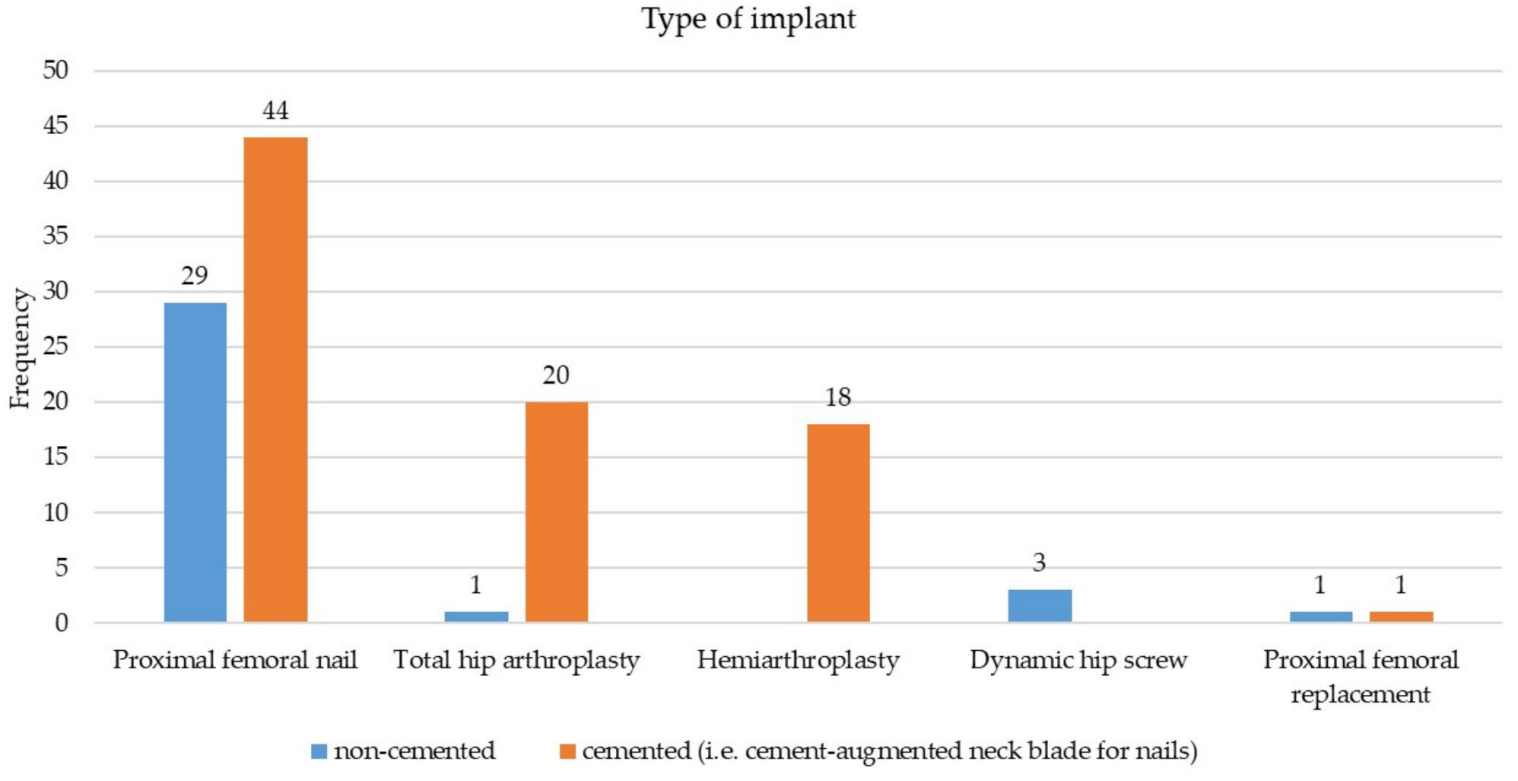
Types of used implants with and without cement augmentation

The Mirels score was determined for all patients by the patient’s history and radiographic imaging. Ten patients were treated bilaterally (S, *n* = 1; S + RT, *n* = 2; RT, *n* = 2). One male and three female patients received surgery alone on one side and adjuvant radiotherapy on the other, 1 female patient received surgery alone on one side and radiotherapy alone on the other side. 2 male patients received radiotherapy alone bilateral. 1 male and 1 female patient received surgery and adjuvant radiotherapy on both sides. 1 male patient was treated by surgery alone bilaterally. Osteolysis was present in 90 cases (male/female: 52/34, 4 patients bilaterally). Prophylactic stabilization for impending fractures was performed in 49 cases (male/female: 27/20, 2 bilaterally, mean Mirels score: 9.7 ± 1.1). In 62 cases surgery was performed due to pathological fracture (male/female: 25/35, 2 bilaterally). A girdlestone situation was reached in 2 patients with high age, very poor prognosis, and inflammatory soft tissue disorders at the same leg. Manifest and impending per- and subtrochanteric fractures were most commonly treated with proximal femoral nail antirotation (PFNA, Johnson & Johnson Medical GmbH, DePuy Synthes, Norderstedt, Germany), (manifest/impending fracture *n* = 22/38). Megaprosthetic reconstructions using tumor endoprosthesis (*n* = 2, Mega C prosthesis, Link, Hamburg, Germany and MUTARS, Implantcast, Buxtehude, Germany) were used twice because of complex subtrochanteric fractures with massive soft tissue involvement, consecutive local tumor debulking and segmental metastatic-induced bone loss. The distribution of all implants and the use of cement augmentation is shown in Fig. 1. The proximal femoral nail section included PFNA (*n* = 60), Gamma-nail (*n* = 4; Stryker, Duisburg, Germany), Targon ® Nail (*n* = 4); Braun, Melsungen, Germany).

Postoperative irradiation was performed after completed wound healing, usually 3 weeks postoperatively at the earliest start date. The postoperative or stand-alone palliative radiotherapy was carried out with a linear accelerator using photon energies of 6 or 15 MV. The fractionation schedules applied consisted of either 10 fractions of 3 Gy over the course of 14 days, 5 fractions of 5 Gy on 5 consecutive weekdays or every other day, or one single fraction of 8 Gy.

### Survival analysis

**Fig. 2:**
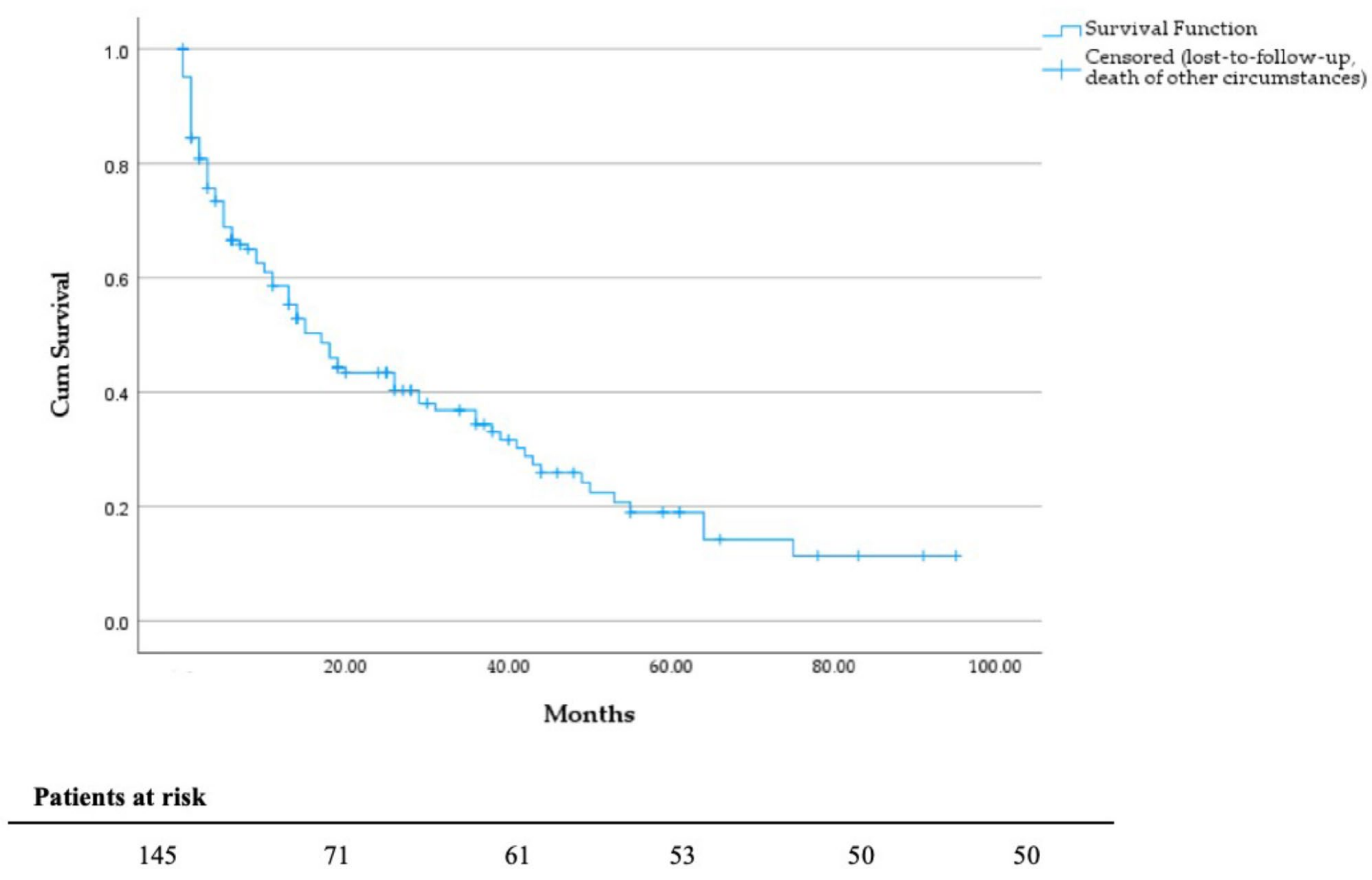
DSS in months, independent of groups

**Fig. 3:**
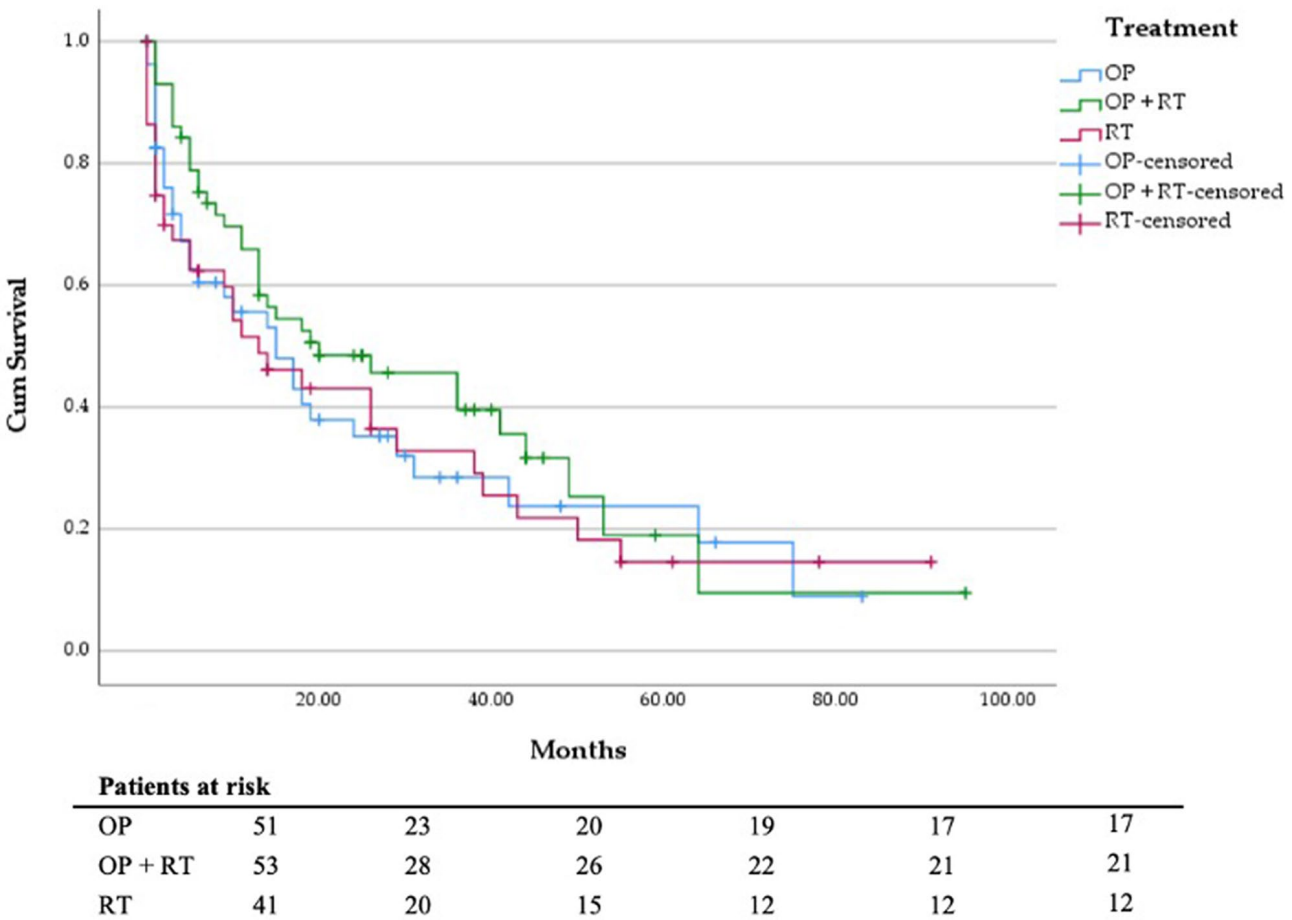
DSS in months depending on local treatment

**Fig. 4:**
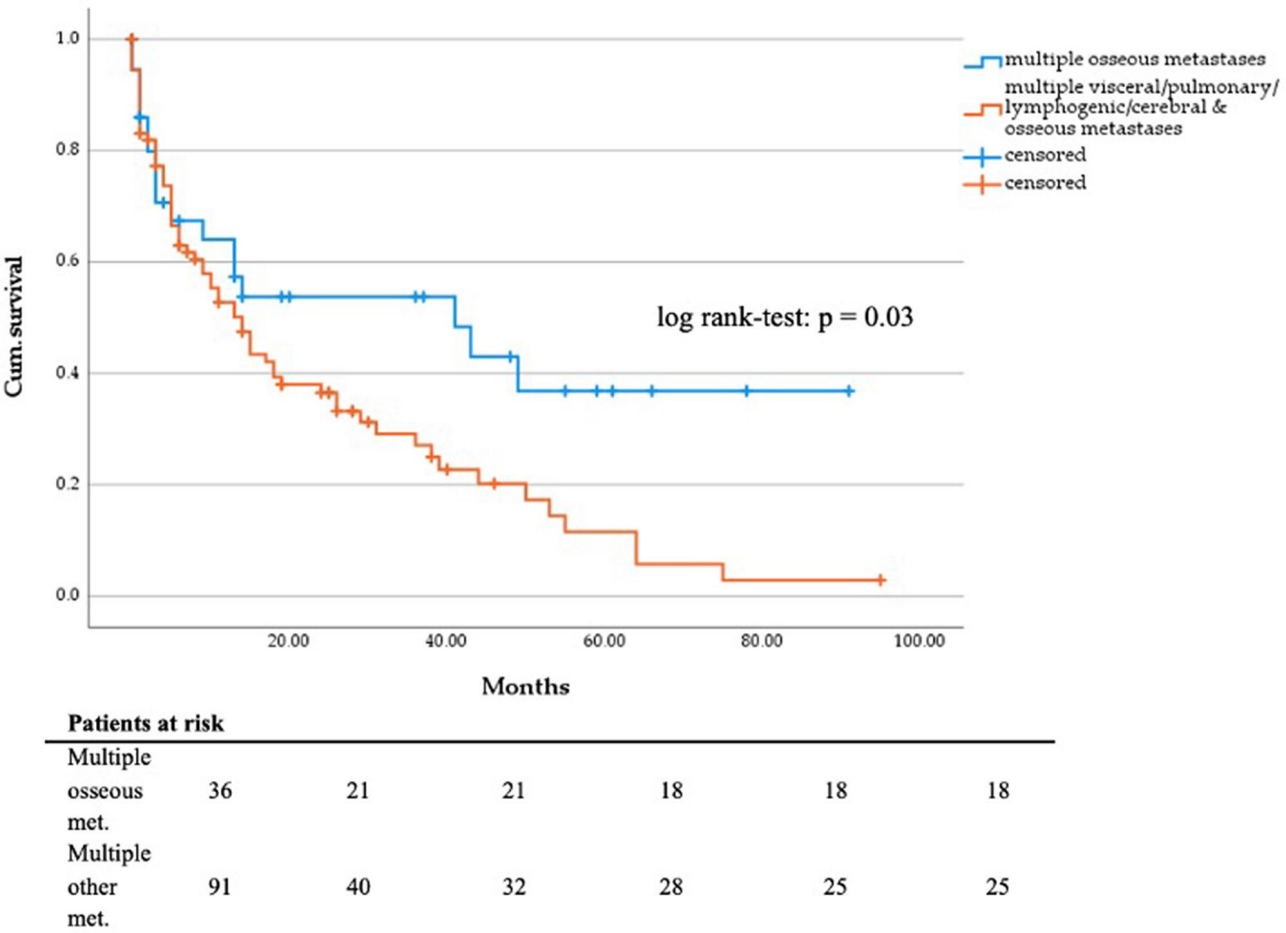
DSS in months regarding patients suffering from multiple osseous (*n* = 91) vs. visceral/pulmonary/lymphogenic/cerebral/osseous metastases (*n* = 36)

Survival analysis revealed that most patients died from their tumor disease (*n* = 101). Tumor-related complications included ileus, paraneoplastic thromboembolic events, and local tumor invasion. The mean follow-up time was 18.2 months with a follow-up rate of 92%. According to the electronic patient file, 91 (62.8%) patients received palliative systemic therapy, e.g. chemotherapy, targeted agents or immunotherapy. Regarding ECOG status, chi-square test showed a significant difference between the groups (Table 1). According to the Mann-Whitney-U-Test, there was a significant difference between the S and RT (*p* = 0.002) and between the S + RT and RT groups (*p* < 0.001). However, given that in the RT group, ECOG status could not be evaluated in 17 patients due to missing documentation, the comparison is, in our opinion, inadmissible and not relevant (Fig. 2). The outcome data and survival analysis according to treatment group and tumor entity are shown in Tables 2, 3, 4 and 5. The comparison of DSS between the S, S + RT and RT groups did not show statistical significance (*p* = 0.765) (Fig. 3). The mean survival of patients treated by hip replacement was 35.4 months (95% CI, 21.2–49.6 months), and that of patients with intramedullary nailing was 30.0 months (95% CI, 21.6–38.5 months). In view of underlying tumor entities and complex fractures patients suffering from breast carcinoma had a significant longer survival than most other entities, e.g. urothelial carcinoma (*p* = 0.001), bronchial carcinoma (*p* = 0.001) and CUP adenocarcinoma (*p* = 0.028). Patients with multiple visceral, pulmonary, lymphogenic, cerebral and osseous metastases (*n* = 91; mean survival = 23.4; 95% CI 17.5–29.3 months) have a significant poorer survival than patients with only multiple osseous metastases (*n* = 36; mean survival = 44.6; 95% CI, 29.9–59.3 months; *p* = 0.03; Fig. 4). The Cox regression analysis showed an influence of gender (*p* = 0.038) and the occurrence of visceral metastases (*p* = 0.009) on DSS (Table 6). The result from the multivariate analysis/Cox-regression that women have a 60% lower and significantly decreased risk of dying from the tumor disease has to be interpreted with caution, as a high-number of female breast cancer patients with favorable DSS have been included. In addition, the occurrence of visceral metastases in carcinoma and sarcoma double the risk of death. Cox regression analysis of the individual entities and metastatic patterns was not possible due to the heterogeneous group distribution in terms of the number of cases.

**Table 2:** Outcome data by therapy group

Outcome		All *n* = 145 (%)	S *n* = 48 (%)	S + RT *n* = 55 (%)	RT *n* = 42 (%)
Status of last follow up	AWD	27 (18.6)	9 (18.8)	13 (23.6)	5 (11.9)
	DOD	95 (65.5)	30 (62.5)	35 (63.6)	30 (71.4)
	DOO	11 (7.6)	6 (12.5)	2 (3.6)	3 (7.1)
	DOC	3 (2.6)	2 (4.2)	1 (1.8)	
	Not specified	9 (6.2)	1 (2.1)	4 (7.3)	4 (9.5)

*Abbreviations* AWD = alive with disease, DOD = died of disease, DOC = died of complications, DOO = died of other circumstances

**Table 3:** Outcome data by tumor entity

Primary tumor site	AWD *n* = 27 (%)	DOD *n* = 95 (%)	DOC *n* = 3 (%)	DOO *n* = 11 (%)	Not specified *n* = 9 (%)
Breast carcinoma (*n* = 31)	6 (22.2)	19 (20.0)	1 (33.3)	3 (27.3)	2 (22.2)
Renal cell carcinoma (*n* = 15)	3 (11.1)	11 (11.6)		1 (9.1)	
Urothelial Carcinoma (*n* = 3)		2 (2.1)			1 (11.1)
Prostate carcinoma (*n* = 27)	8 (29.6)	12 (12.6)		5 (45.5)	2 (22.2)
Bronchial Carcinoma (*n* = 27)	4 (14.8)	22 (23.2)			1 (11.1)
CUP adenocarcinoma (*n* = 6)	1 (3.7)	2 (2.1)			3 (33.3)
Esophageal carcinoma (*n* = 3)		3 (3.2)			
Colon carcinoma (*n* = 1)				1 (9.1)	
Rectal carcinoma (*n* = 3)		3 (3.2)			
Thyroid carcinoma (*n* = 1)		1 (1.1)			
Laryngeal carcinoma (*n* = 1)		1 (1.1)			
Rhabdomyosarcoma (*n* = 2)		2 (2.1)			
Leiomyosarcoma (*n* = 1)		1 (1.1)			
Angiosarcoma (*n* = 2)		2 (2.1)			
Multiple myeloma (*n* = 15)	2 (7.4)	10 (10.5)	2 (66.7)	1 (9.1)	
B-cell lymphoma (*n* = 2)	1 (3.7)	1 (1.1)			
Chronic lymphocytic leukemia (*n* = 1)	1 (3.7)				
Malignant melanoma (*n* = 1)	1 (3.7)				
Penile carcinoma (*n* = 1)		1 (1.1)			
Hepatocellular carcinoma (*n* = 1)		1 (2.1)			
Gastrointestinal stromal tumor (*n* = 1)		1 (1.1)			

*Abbreviations* AWD = alive with disease, DOD = died of disease, DOC = died of complications, DOO = died of other circumstances

**Table 4:** Disease specific survival (DSS) by treatment group

	All *n* = 145	S *n* = 48	S + RT *n* = 55	RT *n* = 42
DSS (months)	29.8 ± 3.1	26.8 ± 4.7	32.2 ± 4.8	27.1 ± 3.0
90% CI (months)	23.8–35.8	17.6–36.0	22.8–41.7	23.7–35.4
6 months DSS (%)	66.6	60.4	75.3	62.4
12 months DSS (%)	58.6	55.6	65.9	51.5

**Table 5:** Disease specific survival (DSS) by tumor entity

Primary tumor site	Mean survival (months)	1 month (%)	6 months (%)	12 months (%)	24 months (%)	36 months (%)
Breast carcinoma (*n* = 31)	26.3	95.5	77.3	72.7	40.9	22.7
Renal cell carcinoma (*n* = 15)	18.1	90.0	45.5	9.1	0	0
Urothelial Carcinoma (*n* = 3)	1.3	50.0	0	0	0	0
Prostate carcinoma (*n* = 27)	25.4	83.3	66.7	41.7	16.7	8.3
NSCLC (*n* = 25)	11.7	62.5	33.3	16.7	12.5	4.2
SCLC (*n* = 2)	1.0	100	0	0	0	0
CUP adenocarcinoma (*n* = 6)	6.2	50.0	50	0	0	0
Esophageal carcinoma (*n* = 3)	4.7	33.3	33.3	0	0	0
Colon carcinoma (*n* = 1)	1.0	100	0	0	0	0
Rectal carcinoma (*n* = 3)	7.7	66.7	33.3	33.3	0	0
Thyroid carcinoma (*n* = 1)	50.0	100	100	100	100	100
Laryngeal carcinoma (*n* = 1)	1.0	100	0	0	0	0
Rhabdomyosarcoma (*n* = 2)	9.5	50.0	50.0	50.0	0	0
Leiomyosarcoma (*n* = 1)	44.0	100	100	100	100	100
Angiosarcoma (*n* = 2)	5.5	50.0	0	0	0	0
Multiple myeloma (*n* = 15)	20.7	90.0	80.0	70.0	30.0	10.0
B-cell lymphoma (*n* = 2)	42.0	100	50	50	50	50
Chronic lymphocytic leukemia (*n* = 1)	34.0	AWD	-	-	-	-
Malignant melanoma (*n* = 1)	20.0	AWD	-	-	-	-
Penile carcinoma (*n* = 1)	3.0	100	0	0	0	0
Hepatocellular carcinoma (*n* = 1)	0.0	0	0	0	0	0
Gastrointestinal stromal tumor (*n* = 1)	1.0	100	0	0	0	0

*Abbreviations* AWD = alive with disease

**Table 6:** Univariate (Kaplan-Meier) and multivariate regression (Cox regression) analysis regarding DSS

	Univariate regression (log rank)	Multivariate regression (Cox regression)
Age ≥ 80 years	*p* = 0.087	*p* = 0.094
Sex	*p* = 0.128	***p*** = 0.038*
Operation on both sides	*p* = 0.69	-
Impending vs. manifest fracture	*p* = 0.72	-
Intramedullary nailing vs. arthroplasty	*p* = 0.429	-
Visceral metastases	***p*** = 0.087*	***p*** = 0.009*
Pulmonary metastases	*p* = 0.393	-
Lymphogenic metastases	*p* = 0.645	-
Cerebral metastases	*p* = 0.268	-
Complications	*p* = 0.136	*p* = 0.188

**Fig. 5:**
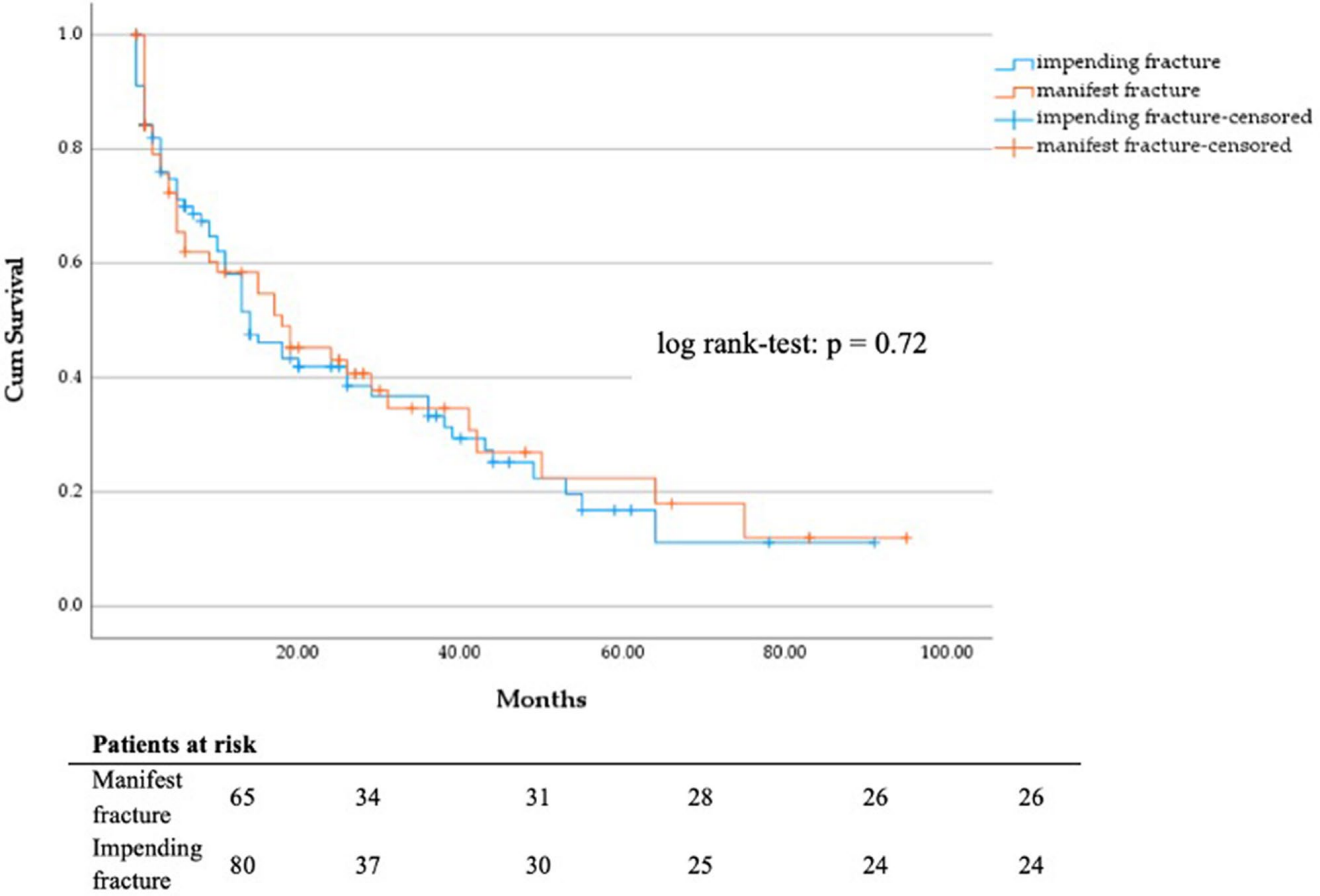
DSS in months regarding manifest (*n* = 65) vs. impending fracture (*n* = 80)

**Fig. 6:**
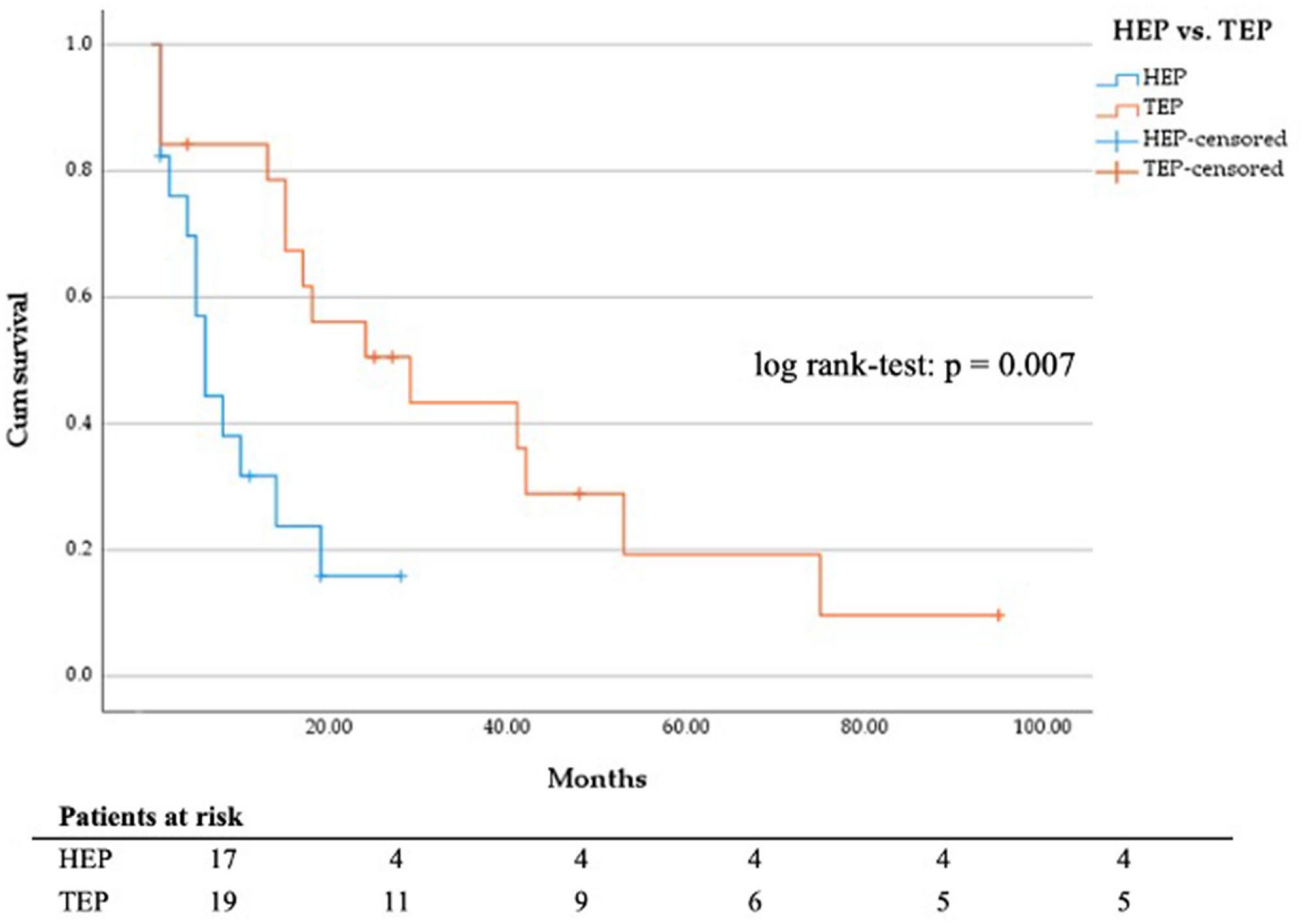
DSS in months regarding hemi hip endoprosthesis (*n* = 17) vs. total hip endoprosthesis (*n* = 19)

No significant difference in DSS of patients with manifest vs. impending fracture (Fig. 5) and of patients treated with total hip arthroplasty and hemiarthroplasty was seen (Fig. 6). Most patients treated with hemiarthroplasty (*n* = 17) suffered from bronchial (*n* = 5) or prostate carcinoma (*n* = 4). Patients who initially received a total hip arthroplasty (*n* = 19) mainly suffered from breast cancer (*n* = 8) or multiple myeloma (*n* = 4).

### Mirels’ score

In patients who received prophylactic surgical treatment the mean Mirels’ score was 9.7 ± 1.1 points. The mean score of patients with radiotherapy alone was 8.1 ± 1.3 points, just at the threshold for recommended surgical treatment. As expected, the groups S (*p* < 0.001; Mann-Whitney-U-Test) and S + RT (*p* < 0.001, Mann-Whitney-U-Test) had a significantly higher Mirels Score than the RT group.

### Complications

Complications occurred in 25 of 155 cases (16.1%) after a mean interval of 9.9 (0–39) months (Table 7). One patient suffered 2 complications. In one case of pathological fracture the oncological treatment had priority because of initial diagnosis of thyroid carcinoma, the patient was operated following completion of the radioiodine-therapy. Another 2 patients with impending and manifest fracture did not undergo operation because they required immediate chemotherapy due to their aggressive and very advanced tumor disease. In the S, S + RT and RT group, complications occurred after a mean period of 7.8 (0–29) months, 17.3 (2-39) months and 6.7 (0–24) months. Complications occurred significantly earlier in the RT group compared to the S + RT group (Table 8). Five patients underwent primary surgery at external hospitals and were referred to us for reoperation due to complications.

**Table 7:** Frequencies (*n*) of complications in different treatment groups

	S *n* = 53 (%)	S + RT *n* = 58 (%)	RT *n* = 44 (%)
Intraoperative bleeding	1 (1.9)		
Infection	3 (5.7)		
Nonunion	2 (3.8)	1 (1.7)	
Implant failure	2 (3.8)	4 (6.9)	
Local progression of metastasis	2 (3.8)		1 (2.3)
Pathological fracture			6 (13.6)
Interprosthetic fracture		1 (1.7)	
Massive increase in pain			2 (4.5)

**Table 8:** Statistical analysis of the occurrence of complications

	S	S + RT	RT
S		*p* = 0.073 (7.8/17.3 months)	
S + RT			***p*** = 0.043* (6.7/17.3 months)
RT	*p* = 0.573 (7.8/6.7 months)		

Occurrence of complications (incl. mean interval) after initial therapy (Mann-Whitney-U-Test, *significant in the 95% confidence interval)

#### Operation (S) group

3 patients had revision surgery due to early infection (1x PFNA, 2x total hip endoprosthesis). Two patients were treated with MUTARS® endoprosthesis due to a nonunion 8 and 23 months after external Targon® nail osteosynthesis for subtrochanteric fracture. Total hip arthroplasty was done in one patient suffering femoral neck screw cut-out after external surgery with Gamma-nail. Two patients suffered local progression, following local radiotherapy after 29 months in 1 patient. One patient got a long stem hip prosthesis after a peri-implant femoral neck fracture with PFNA after 7 months.

#### Operation + adjuvant radiotherapy (S + RT) group

One patient had total hip arthroplasty after suffering from nonunion 16 months after external Targon® Nail implantation. 3 patients received hip arthroplasty due to blade cut-out after 2 and 16 months (1x Gamma-nail & 1x DHS ex domo, 1x PFNA in domo). 2 patients had implant breakage 13 and 18 months postoperatively. Implant removal and femoral head resection was done once due to impaired general conditions while proximal femoral replacement was done for the other one. Interprosthetic fracture was seen in one patient with PFNA and knee arthroplasty after 39 months.

#### Radiotherapy (RT) group

Six patients developed pathological fractures one day, 4 days, 1 (2x), 4 and 9 months after initial radiotherapy (13.6%). Seen retrospectively their mean Mirels score before the start of radiation was 9.5 (8–11) months, an indication for surgical treatment. Depending on their individual fracture localization these 6 patients were treated by PFNA implantation or hip arthroplasty. Another two patients experienced an exacerbation of pain 3 and 6 months after initial treatment and therefore were operated. One patient had progression of local metastasis 24 months after local radiotherapy and was then re-irradiated.

**Fig. 7:**
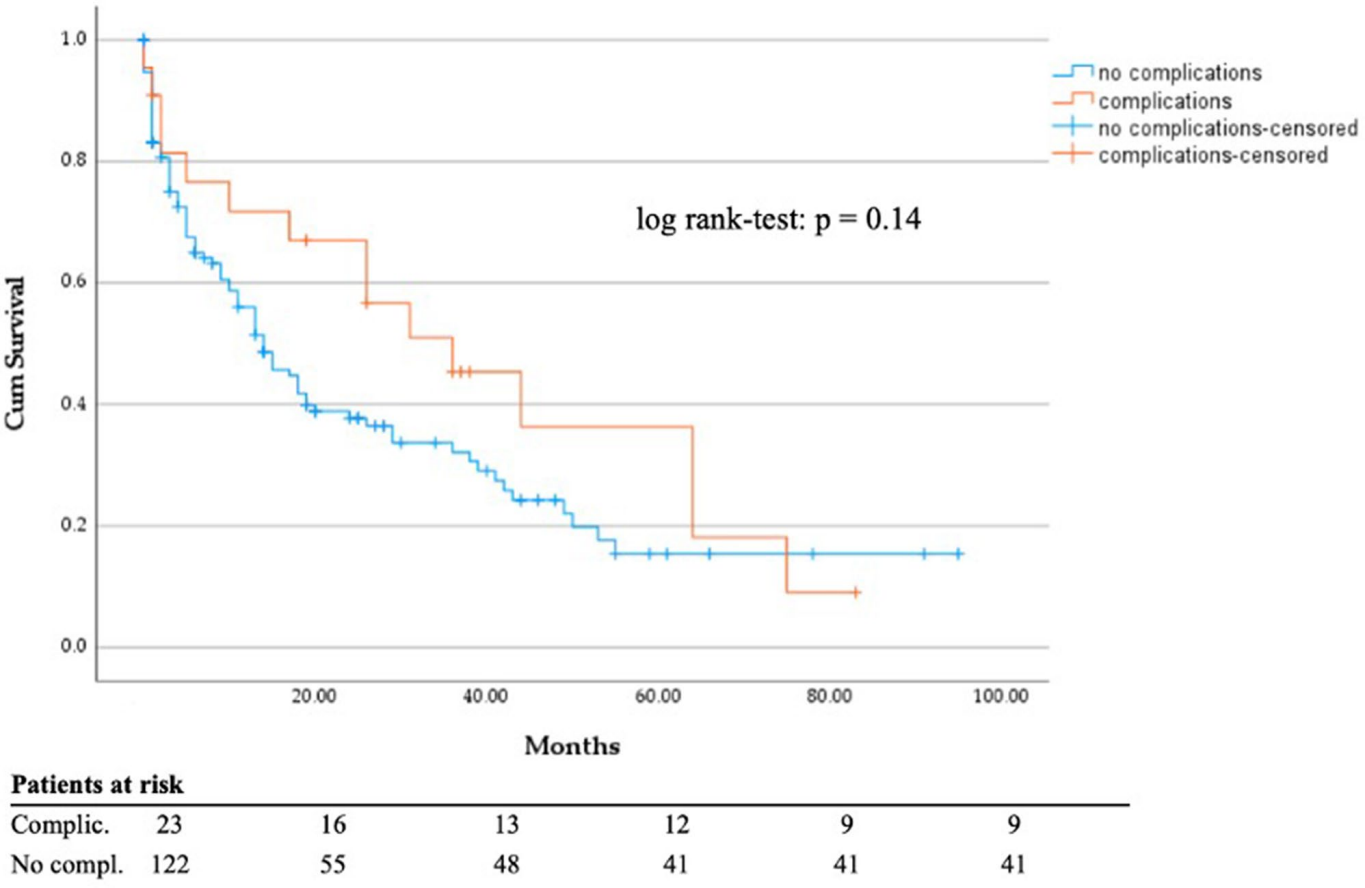
DSS regarding complications vs. no occurrence of complications

**Fig. 8:**
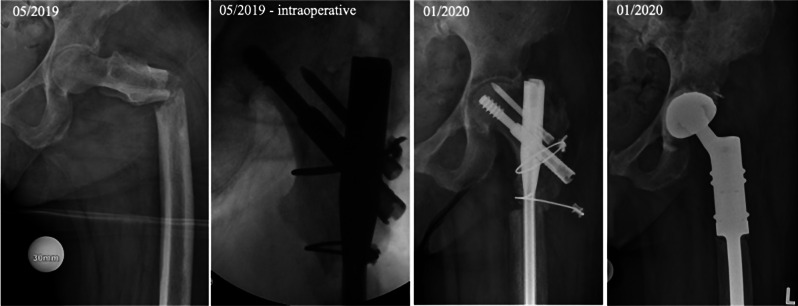
Failure osteosynthesis

**Fig. 9:**
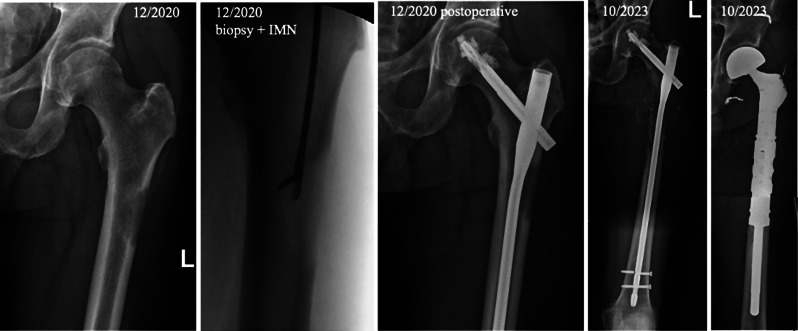
Implant failure due to progressive metastasis

Chi-square test showed no statistically significant correlation between all 3 groups (S, S + RT, RT) regarding the occurrence of complications (χ²(2) = 2.337, *p* = 0.31). There was also no significant difference in DSS between patients with and without complications (*p* = 0.14; Fig. 7). Comparison of complications between patients treated with total hip arthroplasty versus hemiarthroplasty showed no significant differences (χ²(1) = 0,89, *p* = 0.35). In all 17 patients treated by hemiarthroplasty no early infection was found. 2 patients developed infection after total hip arthroplasty group. There was also no significant correlation between occurrence of material failure in patients who were treated with IMN regarding cemented vs. cementless femoral neck blade (χ²(1) = 1.144, *p* = 0.28). Out of the 65 patients treated with PFNA, Gamma nail or Targon® nail, only four experienced a complication. Two examples of material failure are shown in Figs. 8 and 9.

Subtrochanteric nonunion and screw cut out 8 months after cephallomedullary nail fixation (Targon® PFT, external hospital) in a 70 to 80-year-old patient suffering from metastasized breast cancer (initial diagnosis 2019, survival with metastatic disease 2019 to 2021). Definitive treatment using proximal femur replacement.

60 to 70-year-old renal cell carcinoma patient (initial diagnosis 2014, survival with multiple metastatic disease 2014 until now) with implant failure (distal screw breakage) almost 3 years after initial treatment with intramedullary nailing (IMN), biopsy and postoperative radiation. Definitive treatment due to progressive osteolysis with proximal resection and replacement (MUTARS) after preoperative embolization.

## Discussion

Surgical management of skeletal metastatic disease, particularly in anatomic regions that are subjected to tremendous biomechanical stress continues to be a major reconstructive challenge. Primary goals in the treatment of impending and manifest fractures are maintenance and restoration of skeletal stability and consequently mobility, pain reduction and thereby improving quality of life [18]. In the present study we did not find a significant difference in DSS between patients in the S, S + RT and RT group. Our data are in line with previous reports by Maerdian et al. [19] who found a mean survival of 17.5 months in 74 patients with metastatic disease of the femur without a difference between patients with manifest and impending pathologic fractures and between patients with radiotherapy or chemotherapy alone compared with additional surgical therapy. They also could not find a survival benefit of patients who have been treated with radiation or chemotherapy alone versus additional surgical fixation.

Local radiotherapy alone is less invasive, has fewer peri-interventional risks and no anesthetic risks. However, different tumor biologies have variable sensitivities to radiotherapy and chemotherapy [20]. Skeletal metastases of melanoma, thyroid or renal cell carcinoma are more often only moderately sensitive or highly resistant to radiotherapy [21]. But, especially for highly palliative patients, or those who require rapid systemic therapy, radiotherapy is an excellent treatment option as it can be performed simultaneously without any delay, whereas surgery prolongs the start of systemic therapy [22, 23]. However, insufficient biomechanical stability or manifest pathological fracture first requires surgical fixation, in order to restore stability and ambulation as well as address severe associated pain. In our patient cohort 12 (24.5%) of 49 patients who were operated due to an impending fracture died less than 3 months after surgery. Out of these 12 only 9 (18.4%) died due to a progression of the tumor disease. In contrast, after initial surgery 35 (71.4%) and 15 (30.1%) patients survived more than 6 and 24 months. The mean Mirels score of six patients who sustained secondary pathological fracture after radiotherapy was 9.5, indicating that they should retrospectively rather primarily have been treated by surgery due to the high fracture risk of 33% with a Mirels score of ≥ 9 points without any false positive rates [12]. As recommended by Mirels, prophylactic fixation was performed at a Mirels score of ≥ 8 but with a false positive rate of 6% [12]. Taking into account all the data and considerations with regard to the Mirels score it has to be noted that, although it is widely accepted and frequently used in everyday clinical practice, it has already been established in 1989 on the basis of only 38 patients with a total of 78 osseous lesions [12]. Therefore, continuous further development of existing and establishment of new and alternative clinical scores for assessment of risk for pathological fractures due to malignancies is absolutely necessary. Some very recent studies including CT-based data findings, artificial intelligence analysis and machine learning methods show already very promising results [12, 24–26]. Proximal femoral fractures, independent of pathologic or traumatic origin in metastatic and/or geriatric patients show a high patient mortality [27–29]. Saad et al. demonstrated that the occurrence of pathologic fractures in breast, prostate cancer, and multiple myeloma were associated with a decreased survival [27]. As opposed to Katzer et al. and Ward et al. [30, 31], who could demonstrate that patients lived longer after prophylactic stabilization than manifest fracture, our study, Maerdian et al. and Angelini et al. [19, 32, 33] were not able to find a significant difference between these patient groups. In our study the mean survival benefit following impending fracture was only about 1 month. Possibly, using a larger patient collective, a clearer difference could be demonstrated. Considering the patients’ survival in terms of surgical treatment, there were no significant differences in median survival following intramedullary nailing (30 months) compared to arthroplasty (35.4 months) in our study. This observation was also confirmed in a study of Zacherl et al. who showed no significant difference in median survival (12.6 months) in a cohort of 59 patients, as did Lin et al. in a cohort of 86 patients (8.8 months) [33, 34]. Intramedullary nailing for manifest or impending pathological trochanteric fractures is mainly performed because it is a relatively simple, less invasive surgical procedure that is associated with fewer complications and a faster recovery than total or hemiarthroplasty of the hip [31, 35]. Interestingly, Maerdian et al. has related the relatively low complication rate of 1.4% infection and 5.4% hardware failure in a comparable patient cohort with a serious risk profile to the fact that most of their patients did not reach a critical survival time sufficient to cause implant-associated complications. Four of their patients who sustained a hardware failure had a mean survival of 40.8 months (outside of the 95% confidence interval) as compared to 15.4 months of patients without any hardware problems [19]. This lends support to the hypothesis that in patients with large osteolytic lesions or segmental cortical bone loss who are expected to have prolonged survival time, either additional cement augmentation or more generous indications for arthroplasty should be considered in order to prevent hardware complications [36]. Most studies have shown a significant reduction in pain and rapid restoration of postoperative function after endoprosthetic treatment [36–38]. However, implantation of intramedullary interlocking simply bridges the osteolytic segment. Therefore, palliative intramedullary nailing should always be accompanied by postoperative radiation for local adjuvant tumor therapy. Due to the poor healing tendency of pathological fractures, patients with long survival have a higher risk to experience late complications such as pseudarthrosis or material fatigue-associated implant failure [39–45]. In our study patients receiving total hip arthroplasty had a significantly longer DSS than those who received hemiarthroplasty. Regarding the frequency of different tumor entities in those groups, patients with breast carcinoma, i.e. a biologically favorable tumor entity, and patients with multiple myeloma, which can be oncologically treated very well with chemotherapy, were initially treated comparably more often with a total hip arthroplasty. The decision to implant a total hip endoprosthesis in the metastatic tumor stage is therefore based on the individual tumor entity, the response to systematic oncological therapy and the general condition of the patient, which is more detailed reflected by the use of ECOG status or Katagiri score. We do not consider the fact that female gender showed a survival advantage in our study to be of sufficient value, as over 1/5 of the patients were female breast cancer patients, who, as mentioned above, also lived significantly longer than most patients with other tumor entities. We were able to show that patients with multiple but only skeletal metastases lived longer than those who also suffered from visceral, pulmonary, lymphogenic or cerebral metastases. This has also been shown in other studies [46–49]. This leads us to screen out patients with exclusively osseous metastases, especially in surgical treatment, preferring joint replacement, which is safer and more stable in the long term. In addition, regarding the negative influence of visceral metastases on DSS, it should be considered whether a complete resection of the bone lesion and reconstruction using a total endoprosthesis is advisable in patients without multiple visceral metastases and thus with a higher life expectancy, while in patients with multiple visceral metastases and an expected lower life expectancy, simple, rapid treatment using nailing is sufficient to maintain quality of life. However, in long time survivors and patients suffering from multiple skeletal metastatic disease of comparably biologically more favorable tumor entities without any lesion in visceral organs or other sites, local control using radiographic/ CT- and MRI-methods should be absolutely considered. In these patients the limb function, stage of fracture consolidation and quality of life should be studied using clinical, radiographic follow-up visits and patient recorded outcome measures (PROMS). Most research groups assume a postoperative survival > 12 months as long survival in case of metastasized disease [50–53]. New and more detailed scoring systems have been proposed in order to identify and stratify those patients for more extensive surgical interventions [54]. There are already several models, such as the OPTIModel, SPRING13 model, PATHFx or the modified Bauer Score as well as the Katagiri Score, which have been developed in order to assist in decision making for the type of overall treatment, but also for the extent of surgical therapy of skeletal metastatic disease, strictly guided by the as precisely as possible estimated patient`s overall life expectancy [51, 52, 55–58]. While the tumor entity is taken into account in almost all models, there is unfortunately no current consensus on other variables (laboratory values, age, visceral metastases, ECOG status [50, 56, 57]. Performance status decisively determines outcome and is included in prognostic scores, e.g. the Katagiri scoring, which comprises six prognostic factors (primary lesion, visceral or cerebral metastases, abnormal laboratory data, poor performance status, previous chemotherapy, multiple skeletal metastases). It allows stratification of patients in risk groups which were found to be significantly associated with survival in the multivariate analysis of 808 patients.

The fact that patient records and the retrospective study design did not allow for complete collection of all these necessary data to complete this score has to be clearly considered a major restriction and limitation of our study.

If an intralesional treatment with prophylactic stabilization or nail osteosynthesis of a pathological fracture is performed, it should always be done with the highest possible level of stability, i.e. placement of all available screws, cement augmentation of the blade and postoperative irradiation for local tumor control. Although all the cut-outs were found in non-cemented of femoral neck screws/ blades, no significant difference in the frequency of complications for intramedullary nailing with or without cement augmentation and in comparison with arthroplasty, was found. We found similar complication rates regarding intramedullary nailing and arthroplasty like Sarahrudi et al. for a comparable patient cohort, underscoring the validity of our results [59]. Lesions close to the joint (femoral neck, femoral head) are addressed with arthroplasty while inter- / subtrochanteric lesions are commonly treated by cephalomedullary interlocking nails. On the other hand, Steensma et al. argued that inter- to subtrochanteric fractures should also be treated with endoprostheses, as fewer revisions would be necessary [45]. Only 1 of 7 patients with implant failure in our study was primarily treated by arthroplasty. We have performed revision surgery for patients with failure of intramedullary nail fixation and progressive osteolysis intertrochanteric non-union by performing hip arthroplasty. However, total hip arthroplasty for intertrochanteric fractures is much more invasive and associated with a higher risk profile for postoperative infection, blood loss and/or increased operation time [60]. Even if conversion from nailing to endoprosthesis appears to be simple in the study of Steensma et al. [51] we think that a well-thought-out indication should be made, especially in palliative situation, to avoid unnecessary large surgeries and increased risk profile, achieve maximum stability, resilience and thus quality of life with a single ultimate surgical treatment. In order to limit the poor healing tendency and thus local progression and implant failure, there is a clear indication for postoperative radiotherapy in all patients with intramedullary nailing [61, 62]. Mid- and long-term effects of the different therapy modalities and their combination should be the aim of future studies enrolling higher patient numbers and considering patients’ pre-interventional individual life expectancy. Moreover, type of neo- and adjuvant systemic therapy, dose of applied radiation, survival prognosis scores etc. were not considered in the observations. In future studies, we should include this information as well as the quality-of-life analysis in our evaluations in order to make even more precise, patient-specific recommendations for indication and implant selection.

## Conclusion

In a palliative situation, the combination of surgery and adjuvant radiotherapy appears to be the treatment of choice for patients with impending and manifest proximal femur fractures. Radiotherapy alone is only indicated for stable lesions or extremely frailed patients, who are unsuitable candidates for surgical treatment. The choice of implant must be decided depending on the performance status of the patient, the exact location/size of the instability/fracture, the underlying tumor entity and presence of visceral metastases and finally the estimated life expectancy. For intramedullary nailing maximum stability should be ensured (long cephalomedullary interlocking nails, cement-augmentation of the femoral neck blade/screw). Subsequent radiotherapy reduces the risk of progressive metastatic destruction, secondary implant failure and increased readmission, revision and complication rates. If the survival prognosis is longer, initial endo-/megaendoprosthetic reconstruction should be considered.

## Data Availability

The primary data supporting the reported results can be provided upon request by the corresponding authors.

## Abbreviations

AWD: Alive with disease
CI: Confidence interval
CT: Computer tomography
DOC: Died of complications
DOD: Died of disease
DOO: Died of other circumstances
DSS: Disease specific survival
ECOG: Eastern Cooperative Oncology Group; Assessment of the health condition of patients
FBL: Femoral bone lesions
FBM: Femoral bone metastases
Gy: Gray
MRI: Magnetic resonance imaging
MV: Megavolts
PET-CT/-MRI: Positron emission computed tomography/magnetic resonance imaging
PFNA: Proximal femoral nail antirotation
RT: Radiation only
S: Surgery only
S + RT: Surgery with adjuvant radiation
SRE: Skeletal-related events
